# Radiographic quantification of left atrial size in dogs with myxomatous mitral valve disease

**DOI:** 10.1111/jvim.16073

**Published:** 2021-02-26

**Authors:** Christopher Lam, Brad J. Gavaghan, Fiona E. Meyers

**Affiliations:** ^1^ Cardiology Service Veterinary Specialist Services Brisbane Queensland Australia

**Keywords:** left atrial enlargement, modified‐vertebral left atrial size, pimobendan, radiographic left atrial dimension, vertebral heart size, vertebral left atrial size

## Abstract

**Background:**

In the absence of echocardiography, identification of cardiomegaly via thoracic radiography is a necessary criterion for classification of disease severity in dogs with myxomatous mitral valve disease (MMVD).

**Objective:**

Modified‐vertebral left atrial size (M‐VLAS) facilitates objective radiographic assessment of the left atrium (LA) in 2 dimensions and identifies LA enlargement more accurately than existing methods.

**Animals:**

Sixty‐four dogs with various stages of MMVD and 6 control healthy dogs.

**Methods:**

Retrospective case–control study. Medical records were searched for dogs with varying severity of MMVD. Modified‐vertebral left atrial size, vertebral left atrial size (VLAS), vertebral heart size (VHS), and radiographic left atrial dimension (RLAD) were measured from thoracic radiographs and compared with echocardiographically derived measurements.

**Results:**

Positive correlation to LA/Ao was identified for M‐VLAS (*r* = 0.77, *P* < .001), VLAS (*r* = 0.76, *P* < .001), RLAD (*r* = 0.75, *P* < .001), and VHS (*r* = 0.67, *P* < .001). Receiver operating characteristic analyzes provided an area under the curve of 0.97 (95% CI, 0.94‐1.00) for M‐VLAS, which was superior to VHS (0.90, 95% CI, 0.94‐1.00, *P* = .03) in identifying dogs with LA/Ao ≥1.6. A cut‐off value of ≥3.4 vertebrae using M‐VLAS provided 92.7% sensitivity and 93.1% specificity in predicting LA enlargement.

**Conclusions and clinical importance:**

M‐VLAS, which is superior to VHS, offers an accurate and repeatable way to radiographically identify LA enlargement in dogs with MMVD.

**Abbreviations**
2D: 2‐dimensional
ACVIM: American College of Veterinary Internal Medicine
ANOVA: analyzis of variance
AUC: area under the curve
CHF: congestive heart failure
CI: confidence interval
ICC: intraclass correlation coefficient
LA: left atrial
LA/Ao: left atrial‐to‐aortic root ratio
LVIDdN: normalized left ventricular internal diameter in diastole
MMVD: myxomatous mitral valve disease
M‐VLAS: modified‐vertebral left atrial size
RLAD: radiographic left atrial dimension
ROC: receiver operating characteristic
VHS: vertebral heart size
VLAS: vertebral left atrial size

## 1 INTRODUCTION

Myxomatous mitral valve disease (MMVD) is the most commonly acquired cardiac disease and the most frequent cause of congestive heart failure (CHF) in dogs.^1^, ^2^ Left‐sided cardiac remodeling occurs as disease progresses,^3^, ^4^, ^5^, ^6^ and when eccentric capacity is reached, the subsequent rise in left atrial (LA) pressure creates an increase in pulmonary capillary pressure to precipitate pulmonary edema and congestion.^7^, ^8^, ^9^ As such, LA enlargement is well‐established as a precursor to congestive failure and a strong prognostic indicator for dogs with MMVD.^2^, ^9^, ^10^, ^11^, ^12^ Pimobendan therapy delays the onset of CHF and reduces cardiac deaths in dogs with stage B2 MMVD.^13^ Although echocardiography is recommended for definitive diagnosis and disease staging of MMVD in dogs, it is not necessarily available nor affordable to every dog, especially in the general practice setting. As such, the recently updated American College of Veterinary Internal Medicine (ACVIM) consensus statement for MMVD recommends the use of thoracic radiography, namely the vertebral heart size (VHS) and vertebral left atrial size (VLAS), to identify dogs with stage B2 MMVD when echocardiography is unavailable to identify the cardiac enlargement by which stage B2 is defined.^14^

Various limitations including breed‐associated variations,^15^, ^16^, ^17^, ^18^, ^19^, ^20^ interobserver variability^21^, ^22^, and the influence of respiratory and cardiac cycle^23^, ^24^ are associated with the objective radiographic assessment of global cardiac size with VHS. Vertebral left atrial size^25^ and radiographic left atrial dimension (RLAD)^26^ are described as methods to specifically assess LA size via thoracic radiography in dogs with MMVD using a single‐dimensional measurement. Both methods are positively correlated with echocardiographically‐derived left atrial‐to‐aortic root ratio (LA/Ao). A VLAS of ≥2.5 vertebrae is 87% specific and 67% sensitive in predicting LA/Ao ≥1.6,^25^ whereas an RLAD of ≥1.8 vertebrae is 93.5% specific and 96.8% sensitive in identifying the same LA/Ao.^26^ The simplicity of the VLAS method is countered by its relatively poor specificity and sensitivity, whereas the superior accuracy of the RLAD measure is offset by the complexity of its acquisition. We propose a new and simplistic method for radiographic LA measurement will be superior to previous methods by assessment of LA size in 2 dimensions.

The objectives of this study are therefore (a) to compare the accuracy of VLAS, VHS, and RLAD at identifying LA enlargement (LA/Ao ≥1.6) in dogs with various stages of MMVD and (b) to introduce and compare a modified‐VLAS (M‐VLAS) measurement to existing radiographic methods for identification of LA enlargement. We hypothesize that M‐VLAS is superior to published methods in radiographic identification of LA enlargement given its additional dimension of LA measurement.

## 2 MATERIALS AND METHODS

Electronic hospital records of dogs diagnosed with MMVD between 3 hospital sites of Veterinary Specialist Services (Underwood, Jindalee and Carrara) by the cardiology service from October 2017 to March 2020 were retrospectively reviewed. Dogs that were < 20 kg, with physical examination, 2 or 3 view thoracic radiographs (dorsoventral and right +/− left lateral), and echocardiogram performed within a 24‐hour period were included in the study. Echocardiograms were performed by either a board‐certified cardiologist (FEM/BJG) or a resident (CL) under their direct supervision. Echocardiographic measurements were taken from established 2‐dimensional (2D) views,^27^ and the LA/Ao measured from a standard right parasternal short‐axis view.^28^ Left ventricular internal diameter in diastole (LVIDdN) was normalized to individual body weight.^29^ The diagnosis of MMVD was made based on clinical findings of a left apical systolic heart murmur combined with characteristic morphological mitral valve changes and valvular regurgitation evident on echocardiogram.^4^ Classification of MMVD disease stages was made according to the ACVIM consensus guidelines.^14^ Dogs that were < 1‐year old, dogs with acute chordae tendinae rupture, or concurrent cardiac disease were excluded from the study. Dogs with tricuspid regurgitation with color Doppler regurgitant jet area < 50% of the atrial area and without right heart remodeling were not excluded from the study. Dogs with thoracic radiographs that were malpositioned or where exposure factors precluded clear assessment of thoracic vertebrae were also excluded. Dogs were included as controls if they were < 20 kg, with no heart murmur on physical exam, no cardiac abnormalities detected on echocardiogram, and had at least 2‐view thoracic radiographs (dorsoventral and right lateral) taken within the same 24‐hour period as echocardiographic assessment. Baseline data retrieved from medical records included sex, age, breed, bodyweight, and medications administered at the time of examination.

**FIGURE 1:**
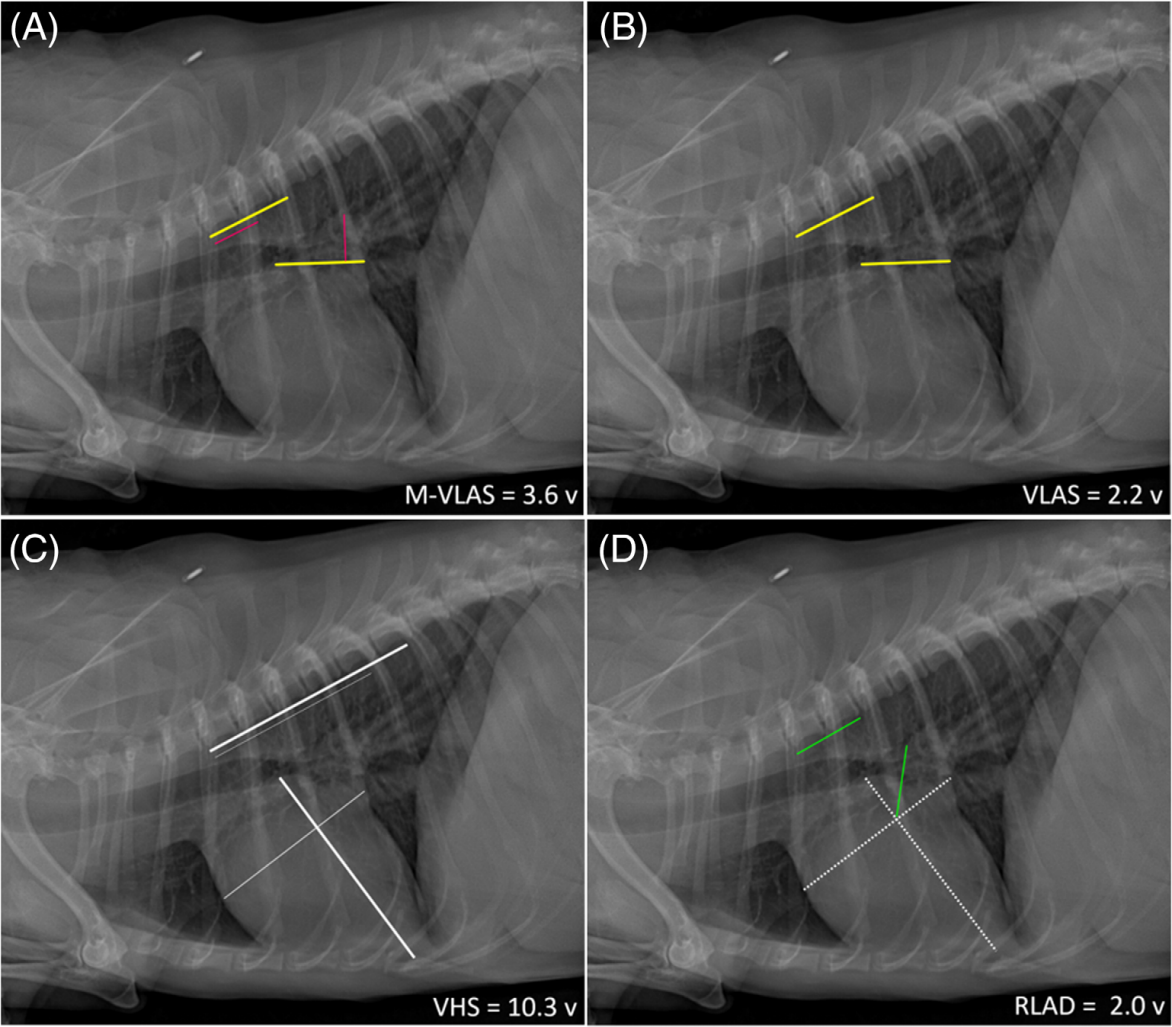
Measurements of M‐VLAS, VLAS, VHS, and RLAD demonstrated on the same right lateral inspiratory radiograph of a dog with stage B2 MMVD. All methods take their respective measurements from the cranial edge of the T4 vertebral body extending caudally, parallel to the vertebral column, rounded to the nearest 0.1 vertebrae. A. MVLAS—an initial line (yellow)—is drawn from the centre of the most ventral aspect of the carina to the intersection between the most caudal aspect of the left atrium and the dorsal border of the caudal vena cava. A second additional line (red) is then drawn from the most distal border of the left atrium towards the first line, intersecting it perpendicularly. Two separate straight lines corresponding to the lengths of the first 2 lines (yellow + red) were then drawn from the cranial edge of the T4 vertebral body extending in a caudal direction, ventral and parallel to the vertebral canal. The M‐VLAS for this dog is 2.2 + 1.4 = 3.6 vertebrae (v). (Note: the first value being the VLAS.) B. Vertebral left atrial size (VLAS = 2.2v)—yellow line C. Vertebral heart score (VHS = 5.7 + 4.6 = 10.3v), vertical axis—thick white line; horizontal axis—thin white line. D. Radiographic left atrial dimension (RLAD = 2.0v)—dashed white lines represent the VHS vertical and horizontal axes, constituting the foundation for the RLAD measurement (green line), which bisects the VHS intersection. *Note* the horizontal axis is always set at the level extending between the cranial cardiac silhouette and the dorsal border of the caudal vena cava for the RLAD measurement, whereas the original VHS method^15^ places the horizontal axis at the widest cardiac width in the central third of the cardiac silhouette regardless of where the caudal vena cava inserts

A right lateral inspiratory thoracic radiograph from each dog was used for all radiographic measurements. Radiographic images were reviewed and measured using a commercial digital radiography viewing software (RadiAnt DICOM viewer, version 5.5.1.23267, Medixant, Poznań, Poland). Vertebral heart size,^30^ VLAS,^25^ and RLAD^26^ were measured according to their respective published methods. Modified‐VLAS was calculated by placement of a digital caliper from the center of the most ventral aspect of the carina and extended to the intersection between the most caudal aspect of the left atrium and the dorsal border of the caudal vena cava as originally described.^25^ A second dimensional measurement was made by placement of the digital caliper at the most distal LA border excluding the pulmonary vein orifice, and extended to perpendicularly intersect with the first line. Two separate straight lines of lengths equal to the measured LA dimensions were then drawn from the cranial edge of the T4 vertebral body extending in a caudal direction, ventral and parallel to the vertebral canal. The M‐VLAS was defined as the sum of the vertebral bodies to the nearest 0.1 vertebrae corresponding to the length of both lines obtained from the left atrium. (Figure 1)

Intraobserver agreement for all 4 radiographic measurements were determined with 1 investigator (CL) performing measurements on 3 separate occasions on 20 dogs randomly selected from the total population. Interobserver variability was determined by 3 investigators (CL, FEM, and BJG) completing all 4 measurement for the same 20 dogs. All investigators were blinded to the identity and disease staging of each dog.

### 2.1 Statistical analyzis

Statistical analyzes were performed using commercial statistical software (GraphPad Prism, version 8.4.1, GraphPad Software, San Diego, California, and MedCalc, version 19.2.0, MedCalc Software, Ostend, Belgium). A *P* value <.05 was considered significant for all analyzes. Distribution of continuous variables was assessed for normality with D'agostino‐Pearson test and for normally distributed variables, values were expressed as the mean ± SD. One‐way analyzis of variance (ANOVA) with Tukey's multiple comparison test was used to compare age, bodyweight, M‐VLAS, VLAS, VHS, and RLAD between control and diseased dogs from various stages. Correlation of LA/Ao with M‐VLAS, VLAS, VHS, and RLAD was assessed using Pearson's correlation. Receiver operating characteristic (ROC) curves, and the area under the curves (AUC) with 95% confidence intervals (CI) using LA/Ao < or ≥ 1.6 as the classifiers were generated for M‐VLAS, VLAS, VHS, and RLAD. Comparison between the AUC of the 4 measurements was performed using Delong's method. Sensitivity and specificity for M‐VLAS, VLAS, VHS, and RLAD were determined with the Youden index to determine the optimal cut‐off value. Inter‐ and intraobserver variabilities were assessed for M‐VLAS, VLAS, VHS, and RLAD via intraclass correlation coefficient (ICC) estimates and their 95% CI based on a single rater, absolute agreement, with 2‐way random (interobserver) and mixed (intraobserver) effect. An ICC value of >0.9 was considered excellent, 0.75 to 0.9 was considered good, 0.5 to 0.75 was considered moderate, and poor if <0.5.^31^

## 3 RESULTS

A total of 70 dogs were included into the study, comprising 64 dogs with MMVD (22 dogs with stage B1, 21 dogs with stage B2 and 21 dogs with stage C) and 6 control healthy dogs. Within the stage B1 group (11 females and 11 males), there were 5 mixed breeds, 4 Cavalier King Charles Spaniels (CKCS), 3 Maltese, 2 Chihuahuas, 2 Dachshunds, 2 Fox Terriers, 2 Poodles, 1 Miniature Schnauzer, and 1 Tibetan Spaniel. Stage B2 dogs (8 females and 13 males) consisted of 6 mixed breeds, 6 CKCS, 2 Poodles, 2 Shih Tzus, 1 Beagle, 1 Chihuahua, 1 Dachshund, 1 Jack Russell Terrier, and 1 Maltese. Within the stage C group (7 females and 14 males), there were 8 mixed breeds, 3 Chihuahuas, 3 Maltese, 2 CKCS, 2 Jack Russell Terriers, 1 Löwchen, 1 Silky Terrier and 1 Toy Poodle. Control dogs (5 females and 1 male) included 1 Alaskan Klee Kai, 1 Fox Terrier, 1 Jack Russell Terrier, 1 Maltese, 1 mixed breed, and 1 Poodle. Table 1 summarizes the descriptive data from all dogs and the medications administered at the time of assessment. Mean age and bodyweight were not significantly different between groups.

**TABLE 1:** Descriptive data for control healthy dogs and dogs with various stages of myxomatous mitral valve disease

Variable	Control	Stage B1	Stage B2	Stage C	*P* value
No. of dogs	6	22	21	21	‐
Female	5	11	8	7	‐
Male	1	11	13	14	‐
Bodyweight (kg)	7.57 ± 2.13	8.37 ± 3.63	9.41 ± 2.93	6.96 ± 3.02	.09
Age (year)	8.72 ± 5.89	11.07 ± 2.77	10.65 ± 2.93	10.96 ± 2.13	.33
LA/Ao	1.26 ± 0.18	1.18 ± 0.14	2.07 ± 0.42^a^	2.39 ± 0.52^a^	< .001
LVIDdN	1.32 ± 0.15	1.51 ± 0.22	2.00 ± 0.18^a^	2.09 ± 0.35^a^	< .001
M‐VLAS	2.60 ± 0.30	2.75 ± 0.55	4.10 ± 0.57^a^	4.57 ± 0.86^a^	< .001
VLAS	1.83 ± 0.29	1.93 ± 0.33	2.66 ± 0.36^a^	2.92 ± 0.54^a^	< .001
VHS	9.28 ± 0.77	10.28 ± 0.76	11.61 ± 0.93^a^	12.04 ± 1.52^a^	< .001
RLAD	1.22 ± 0.24	1.54 ± 0.42	2.34 ± 0.43^a^	2.71 ± 0.66^a^	< .001
Medications at time of assessment	n/a	Frusemide (2/22)^b^, pimobendan (2/22)^b^, codeine (1/22), doxycycline (1/22)	Pimobendan (6/21), trilostane (2/21), clopidogrel (1/21), rivaroxaban (1/21)	Frusemide (13/21), pimobendan (12/21), benazepril (6/21), trilostane (1/21), insulin (1/21), amoxycillin‐clavulanate (1/21), diltiazem (1/21)	‐

*Note*: Means with respective SDs provided for bodyweight, age, LA/Ao, VHS, RLAD, VLAS, and M‐VLAS.

Abbreviations: LA/Ao, left atrium‐to‐aortic root ratio; LVIDdN, normalized left ventricular internal diameter at end diastole; M‐VLAS, modified vertebral left atrial size; RLAD, radiographic left atrial dimension; VHS, vertebral heart size; VLAS, vertebral left atrial size.

^a^ Statistically significant difference compared to control and stage B1 dogs (*P* < .001).

^b^ Medications were subsequently discontinued upon correct disease stage classification.

**FIGURE 2:**
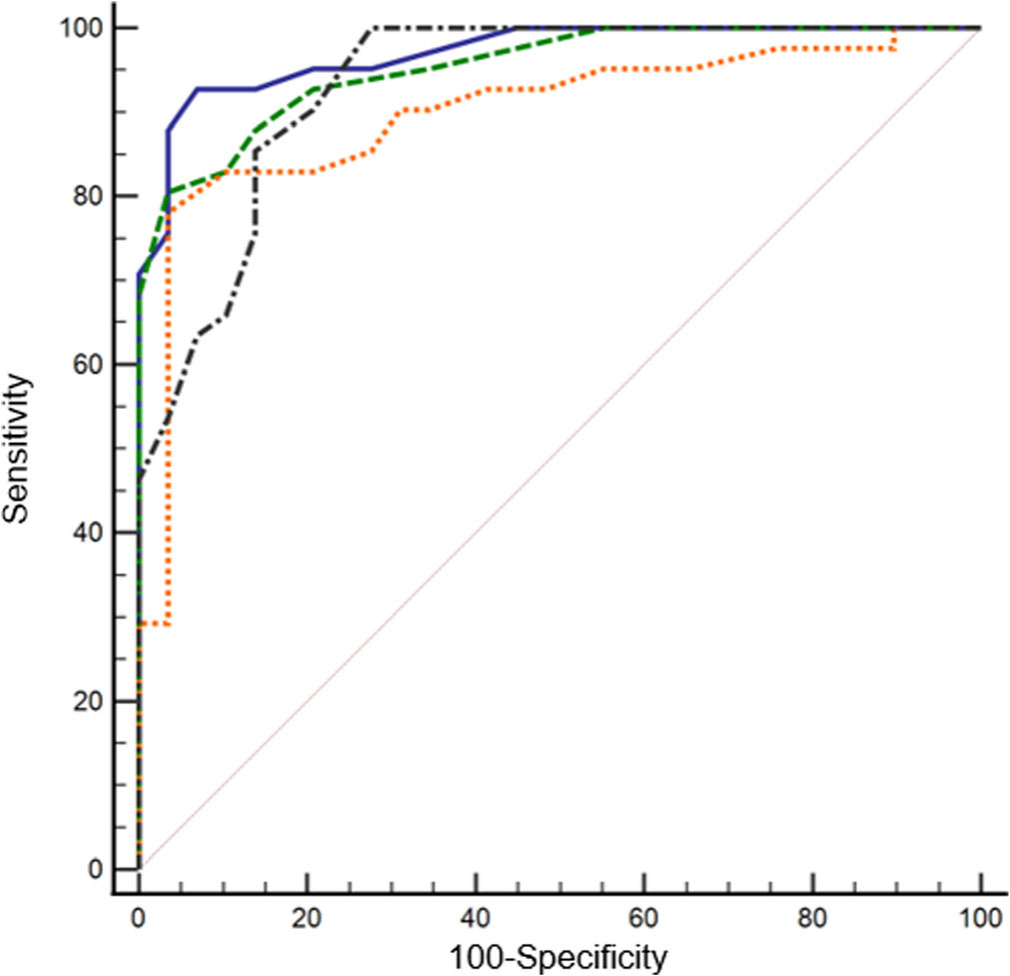
Receiver operating characteristic (ROC) curves of M‐VLAS, VLAS, VHS, and RLAD measured in 29 dogs with LA/Ao < 1.6, and 41 dogs with LA/Ao ≥1.6. Significant difference in AUC was observed between M‐VLAS (AUC 0.97, 95% CI 0.90‐1.00) and VHS (AUC 0.90, 95% CI 0.80‐0.96) (*P* = .03). Blue solid—M‐VLAS, green dashed—VLAS, orange dotted—VHS, and black dot‐dashed—RLAD

Mean M‐VLAS, VLAS, VHS, RLAD, LA/Ao, and LVIDdN were all significantly higher in dogs with stage B2 and C MMVD compared to control dogs and dogs with stage B1 MMVD (*P* < .001). No differences were observed between control and stage B1 dogs, nor between stage B2 and C dogs for any radiographic or echocardiographically‐derived value (Table 1). Left atrial‐to‐aortic root ratio was positively correlated with M‐VLAS (*r* = 0.77, 95% CI 0.65‐0.85, *P* < .001), VLAS (*r* = 0.76, 95% CI 0.64‐0.85, *P* < .001), VHS (*r* = 0.67, 95% CI 0.52‐0.78, *P* < .001), and RLAD (*r* = 0.75, 95% CI 0.63‐0.84, *P* < .001). The ROC analyzes indicated that all radiographic methods were useful to identify dogs with LA enlargement (LA/Ao ≥1.6). Area under the curve for M‐VLAS, VLAS, VHS, and RLAD was 0.97 (95% CI 0.94‐1.00), 0.95 (95% CI 0.91‐1.00), 0.90 (95% CI 0.80‐0.96), and 0.93 (95% CI 0.85‐0.98), respectively. A cut‐off value of ≥3.4 vertebrae using the M‐VLAS method provided 92.7% sensitivity and 93.1% specificity (Youden index 0.86). Comparatively, a VLAS cut‐off value of ≥2.4 vertebrae provided 80.5% sensitivity and 96.6% specificity (Youden index 0.77). For VHS, a cut‐off value of ≥11.1 was 78.1% sensitive, and 96.6% specific (Youden index 0.75). Lastly, a cut‐off value of ≥1.7 using RLAD provided a sensitivity of 100% and specificity of 72.4% (Youden index 0.72). The AUC was significantly greater for M‐VLAS (0.97, 95% CI 0.90‐1.00) compared to VHS (0.90, 95% CI 0.80‐0.96) (*P* = .03). The AUC was similar for other radiographic methods. (Figure 2).

Both intra‐ and interobserver variabilities assessed by ICC demonstrated good to excellent agreement for all radiographic methods (ICC > 0.75, *P* < .001) (Table 2).

**TABLE 2:** Intra‐ and interobserver agreement for modified vertebral left atrial size (M‐VLAS), vertebral left atrial size (VLAS), vertebral heart size (VHS) and radiographic left atrial dimension (RLAD)

	Intraobserver agreement			Interobserver agreement		
Methods	ICC	95% CI	*P* value	ICC	95% CI	*P* value
M‐VLAS	0.88	0.77 to 0.95	< .001	0.93	0.85 to 0.97	< .001
VLAS	0.97	0.94 to 0.99	< .001	0.92	0.84 to 0.96	< .001
VHS	0.99	0.98 to 1.00	< .001	0.94	0.87 to 0.97	< .001
RLAD	0.97	0.95 to 1.00	< .001	0.90	0.81 to 0.96	< .001

Abbreviations: CI, confidence interval; ICC, intraclass correlation coefficient.

## 4 DISCUSSION

The objectives of this study were to examine and compare different radiographic methods for identification of echocardiographically measured LA enlargement (LA/Ao ≥1.6) and to introduce a modified‐VLAS method that aimed to incorporate an additional dimension in measuring LA size on radiographs. The results of this study showed a positive correlation between M‐VLAS, VLAS, VHS and RLAD with LA/Ao in dogs with MMVD. Significantly higher M‐VLAS, VLAS, VHS, and RLAD were observed in stage B2 and C dogs than stage B1 and control healthy dogs. Optimal cut‐off values generated from ROC curves indicated that a M‐VLAS of ≥3.4 vertebrae offered 93% sensitivity and specificity in predicting significant LA enlargement as defined by a LA/Ao ≥1.6. Comparison of ROC curves indicated M‐VLAS was superior to VHS in identifying this LA enlargement.

The original VLAS method aimed to quantify LA size radiographically based on a single‐dimensional measurement that roughly represented the transverse diameter of the LA body. Yet, LA functional anatomy and geometry is complex, with the left atrium described as a cylinder with an almost fixed head and distensible walls attached to a piston (ie, mitral annulus).^32^ The process of LA remodeling is equally complex and non‐uniform.^33^ Recognizing the limitations of only using a single 2D lateral radiograph to quantify the complex LA anatomy, the M‐VLAS method aimed to complement the existing VLAS method with an additional aspect of measurement, incorporating the dorsoventral dimension of the LA chamber. The goal was to capture dogs with LA remodeling secondary to MMVD that would have otherwise been missed by the VLAS method given its potential limitations as a single‐dimensional measurement. As an example, the dog illustrated in Figure 1 fulfilled the physical exam, radiographic and echocardiographic findings (LA/Ao = 1.90 and LVIDdN = 1.8) of stage B2 disease, thereby meeting the ACVIM consensus statement criteria^14^ for initiating pimobendan therapy. The secondary LA remodeling in this dog however was predominantly in a dorsoventral direction, and only modestly in a transverse direction. As such, the radiographically determined VLAS of 2.2 vertebrae (VLAS ≥2.3 considered abnormal)^25^ failed to identify LA enlargement in this dog. However, by incorporating a second dimensional measurement, the M‐VLAS of this individual (3.5 vertebrae) better captured the full extent of LA enlargement using the cut‐off value of ≥3.4 vertebrae. Within the cohort of stage B2 dogs in this study, it is noteworthy that 2 (9.5%) of 21 dogs were correctly identified with LA enlargement using the M‐VLAS method (≥ 3.4 vertebrae) but categorized as normal when measured with the VLAS method using the published cut‐off of <2.3 vertebrae. In contrast, no stage B2 MMVD dogs with clinically significant LA enlargement detected via the VLAS method were missed by M‐VLAS. Nonetheless, when comparing the AUC of the 2 radiographic methods, no significant difference was observed. This could be due to the small study population being underpowered to detect significant differences in the current study (ie, a type II statistical error). Definitive evaluation would require a larger study with prospective power analyzis directing enrolment numbers.

The results of this study have reinforced the previously described^24^, ^25^, ^34^, ^35^, ^36^ positive correlation between VLAS and LA/Ao (*r* = 0.76, 95% CI 0.64‐0.85, *P* < .001). The optimal cut‐off value of ≥2.4 vertebrae derived from ROC analyzis in the current study to identify a LA/Ao ≥1.6 was close to the cut‐off value of ≥2.3 vertebrae originally proposed,^25^ and the cut‐off value of ≥2.5 vertebrae reported subsequently.^34^, ^36^ Direct comparison however is difficult given previously described methodology^34^ rounded their measurements to the nearest 0.25 vertebrae as opposed to 0.1 vertebrae used by the other studies. A dog with a VLAS of 2.4 using the original published method would therefore be categorized as 2.5 if it is rounded up to the nearest 0.25 vertebral units. Similar sensitivity but higher specificity of VLAS (≥ 2.4) has been demonstrated in the current study (81% and 97%) than what has been reported in the previous studies (67%/85%,^25^ 70%/84%,^34^ and 86%/84%^36^) using a VLAS of ≥2.5. The reason for the differences could simply be because of variation in study samples as all studies have demonstrated a good‐excellent ICC for intra‐ and interobserver variability.^25^

One of the most important findings of the current study is the superiority of M‐VLAS to identify LA enlargement compared to VHS. (Figure 2) Both the correlation coefficient of M‐VLAS with LA/Ao was higher than that of VHS (*r* = 0.77 vs. 0.67) and the AUC of M‐VLAS was greater than that of VHS (0.97 [95% CI 0.90‐1.00] vs. 0.90 [95% CI 0.80‐0.96], *P* = .03). This is not a surprising result as VHS is a measurement of global cardiac size whereas M‐VLAS aims to more directly reflect LA size. Interestingly, neither VLAS nor RLAD which similarly aim to represent LA size demonstrated superiority over VHS by ROC analyzis. The additional dimension included in M‐VLAS measurement over the single axis measurement of VLAS and RLAD presumably facilitates a more comprehensive measure of LA size and the statistical distinction between M‐VLAS and VHS but not between VLAS, RLAD and VHS. Minimization of both false negatives and false positives is equally important when ascertaining the optimal measurement technique to radiographically identify cardiomegaly in dogs with MMVD. In the current study, M‐VLAS, with the cut‐off value of ≥3.4 vertebrae, was highly specific and sensitive (93%) at identification of LA enlargement (La/Ao ≥1.6), whereas VHS (cut‐off value of ≥11.1) provided high specificity (97%) at the expense of low sensitivity (78%).

Multiple publications have evaluated the radiographic method to objectively assess heart size by VHS since its introduction in 1995.^37^ The limitations of VHS include multiple breed‐associated variations,^15^, ^16^, ^17^, ^18^, ^19^, ^20^ interobserver variability,^21^, ^22^ and the influence of respiratory and cardiac cycles.^23^, ^24^ While respiratory cycle can be controlled when radiographs are taken, cardiac cycle cannot. The mean VHS ± SD ranges from 9.9 ± 0.8 to 10.4 ± 0.8 vertebrae between end‐diastolic and end‐systolic measurements at peak inspiration for dogs positioned in right lateral recumbency.^24^ On average, mean VHS ± SD is 0.3 ± 0.3 vertebrae greater in diastole than in systole at peak inspiration, with VHS varying up to 0.97 vertebral units over the cardiac cycle in some individuals.^23^ Similar influence of cardiac cycle however is not observed on VLAS.^24^ Without further studies, one can only deduce that M‐VLAS, being a derivative of VLAS, might also show similar lack of influence by cardiac cycle. Interobserver variability is as a potential limitation of VHS. Interobserver variability ranges from a maximum difference of approximately 1.0 vertebral unit^21^ to >1.0 vertebral unit.^22^ Vertebral heart size measurement is not influenced by individual observer clinical experience but is affected by the observer's selection of reference points and transformation of long‐ and short‐axis dimensions into VHS units.^21^ This observation was not evident in the current study as all radiographic methods showed good‐excellent intra‐ and interobserver repeatability.

The RLAD is proposed to assess LA enlargement in dogs with mitral regurgitation.^26^ This method utilizes VHS as its foundation by adding a third line that bisects the longitudinal and horizontal VHS axes at a 45° angle caudodorsally, extending to the most dorsal aspect of the LA bulge. The length of the third line was subsequently measured from T4 vertebral body, providing the RLAD measurement. An RLAD of ≥1.8 vertebrae is 93.5% specific and 96.8% sensitive in detecting LA enlargement. In the current study, an RLAD of ≥1.7 vertebrae provided the best sensitivity (100%) and specificity (72.4%), but a RLAD of ≥1.8 vertebrae as a measure of LA/Ao ≥1.6 in the current study could not replicate a comparable accuracy to the original publication (sensitivity 90.2%, specificity 79.3%). This may be due to the higher proportion of dogs with advanced disease (stage C) in the original study than the current study (60% vs. 50%), with more pronounced LA enlargement (mean LA/Ao 2.47 ± 0.55 vs. 2.23 ± 0.49). Furthermore, many limitations of the VHS measurement may also apply to the measurement of RLAD based on the dependency on VHS to derive RLAD. Moreover, in the original described methodology for RLAD,^26^ the horizontal axis of the heart is fixed to the intersection of the caudal cardiac silhouette and the dorsal border of the caudal vena cava. This deviates from the original VHS measurement method in which the horizontal axis is measured in the central third of the cardiac silhouette across the widest point independent of the vena caval position.^37^ If the original and well‐known VHS method is inappropriately utilized in clinical practice as the foundation for measuring RLAD, it precludes interpretation of the published RLAD of ≥1.8^26^ or ≥ 1.7 (current study) as indicative of LA/Ao ≥1.6.

There is no significant difference between VHS and VHS + VLAS in detection of LA enlargement (as defined by LA/Ao ≥1.6)^35^; however, direct comparison between VHS and VLAS is not reported. This study demonstrated comparable efficacy of VHS and VLAS in identifying echocardiographically detected LA enlargement. Moreover, similar to the original RLAD study,^26^ our results demonstrated comparable efficacy of RLAD and VHS in detection of LA enlargement.

The current study has several limitations. Firstly, because of the retrospective design sample sizes were small especially in the healthy group (n = 6). However, the aim of this study was not to differentiate between healthy and diseased dogs nor to establish normal reference ranges, but rather to assess the efficacy of objective radiographic measurements to identify LA enlargement (LA/Ao ≥1.6), which together with other cardiac criteria, is an indication for pimobendan therapy.^14^ Being a retrospective study, dog recruitment also could not be strictly controlled, and echocardiographic measurements might be subjected to interobserver variability. To reduce the possibility of misclassification, all dogs were assessed and classified by board‐certified cardiologists or a resident under direct supervision by board‐certified cardiologists. In addition, all dogs with heart failure were classified by documentation of clinical signs of dyspnea, radiographic evidence of pulmonary edema, and positive response to diuretic therapy. Secondly, the presence of concurrent systemic disease and medical treatment were not controlled and therefore any secondary change in intravascular volume between when thoracic radiographs and echocardiogram were performed could contribute to discrepancies in cardiac size. However, our inclusion criteria were limited to dogs with echocardiography and thoracic radiographs performed within a 24‐hour period to minimize treatment effects and changes in intravascular volume. Only 1 dog with stage B1 MMVD from the cohort received fluid therapy intravenously during assessment for treatment of pancreatitis. Thirdly, we recognize the limitations of 2D echocardiography in accurately quantifying LA size and volume.^38^, ^39^ Finally, because of its retrospective nature, cases were recruited across 3 Veterinary Specialist Services hospital locations. Individual radiology unit and exposure factors used for each case were consequently different which has the potential to reduce consistency. The current study also included a variety of breeds. The effect of breed variability to the outcome of the various radiographic measurements (especially VLAS, RLAD, and M‐VLAS) is unknown and will likely be the target for future studies.

## OFF‐LABEL ANTIMICROBIAL DECLARATION

Authors declare no off‐label use of antimicrobials.

## CONFLICT OF INTEREST DECLARATION

Authors declare no conflict of interest.

## INSTITUTIONAL ANIMAL CARE AND USE COMMITTEE (IACUC) OR OTHER APPROVAL DECLARATION

Authors declare no IACUC or other approval was needed.

## HUMAN ETHICS APPROVAL DECLARATION

Authors declare human ethics approval was not needed for this study.
